# Evaluation of Antibacterial and Antiviral Drug Effectiveness in COVID-19 Therapy: A Data-Driven Retrospective Approach

**DOI:** 10.3390/pathophysiology29010009

**Published:** 2022-03-07

**Authors:** Rika Yulia, Putri Ayu Irma Ikasanti, Fauna Herawati, Ruddy Hartono, Puri Safitri Hanum, Lestiono, Dewi Ramdani, Abdul Kadir Jaelani, Kevin Kantono, Heru Wijono

**Affiliations:** 1 Department of Clinical and Community Pharmacy, Faculty of Pharmacy, Universitas Surabaya, Surabaya 60293, Indonesia; rika_y@staff.ubaya.ac.id (R.Y.); putriayuirma@gmail.com (P.A.I.I.); 2 Department of Pharmacology and Clinical Pharmacy, Faculty of Pharmacy, Universitas Indonesia, Depok 16424, Indonesia; 3 Department of Pharmacy, Rumah Sakit Bhayangkara, Surabaya 60231, Indonesia; rudd_apt@yahoo.com; 4 Faculty of Medicine, Universitas Surabaya, Surabaya 60293, Indonesia; purisafitrihanum@staff.ubaya.ac.id (P.S.H.); herwyno@staff.ubaya.ac.id (H.W.); 5 Department of Pharmacy, Rumah Sakit Pusat TNI Angkatan Laut (RSPAL) Dr. Ramelan, Surabaya 60244, Indonesia; lestionoapt@gmail.com; 6 Department of Pharmacy, Rumah Sakit Umum Haji, Surabaya 60177, Indonesia; deramdani123@gmail.com; 7 Department of Pharmacy, RSUD Bangil, Pasuruan 67153, Indonesia; abusuquf@yahoo.co.id; 8 Department of Food Science, Auckland University of Technology, Private Bag 92006, Auckland 1142, New Zealand; kevin.kantono@aut.ac.nz

**Keywords:** defined daily dose, COVID-19, antibacterials, antivirals, SARS-CoV-2

## Abstract

The clinical manifestations associated with COVID-19 disease is mainly due to a dysregulated host response related to the overexpression of inflammatory markers. Until recently, only remdesivir had gained FDA approval for COVID-19 hospitalized patients and there are currently no evidence-based therapeutic options or options for prevention of complications that have been established. Some medical treatments such as antivirals, antibacterials, antithrombotics, antipyretics, corticosteroids, interleukin inhibitors, monoclonal antibodies, convalescent plasma, immunostimulants, and vitamin supplements have been utilized. However, there are limited data to support their effectiveness. Hence, this study was attempted to identify and evaluate the effectiveness of antibacterials and antivirals used for COVID-19 using a retrospective cross-sectional approach based on the medical records of adult patients in four hospitals. The number of antibacterials was calculated in defined daily dose (DDD) per 100 bed-days unit. Both mixed-logit regression and analysis of covariance were used to determine the effectiveness of the aforementioned agents in relation to COVID-19 outcome and patients’ length of stay. The model was weighed accordingly and covariates (e.g., age) were considered in the model. Heart disease was found to be the most common pre-existing condition of COVID-19 hospitalized patients in this study. Azithromycin, an antibacterial in the Watch category list, was used extensively (33–65 DDD per 100 bed-days). Oseltamivir, an antiviral approved by the FDA for influenza was the most prescribed antiviral. In addition, favipiravir was found to be a significant factor in improving patients’ COVID-19 outcomes and decreasing their length of stay. This study strongly suggests that COVID-19 patients’ received polypharmacy for their treatment. However, most of the drugs used did not reach statistical significance in improving the patients’ condition or decreasing the length of stay. Further studies to support drug use are needed.

## 1. Introduction

COVID-19 infection cases and mortality in all countries are high, which has raised global awareness. Indonesia is the fourth most populous country in the world, and thus is predicted to suffer greatly and over a longer period compared to other less-populous countries. Indonesia has been greatly affected by COVID-19 with a Case Fatality Rate (CFR) of 8.9% at the end of March 2020 [1] in comparison to the global figure of 7.3% [2]. In Jakarta, the capital city of Indonesia, it has been reported that there were 4265 hospitalised patients with PCR-confirmed COVID-19 in 55 hospitals from the period of March to July 2020; 3768 (88%) were discharged and 497 (12%) died; 5% were children, the median age was 46 years (interquartile range: 32–57 years), and 31% had more than 1 reported comorbidity [3]. COVID therapy management at every hospital in Indonesia was fairly similar. In March 2020, Indonesia’s Ministry of Health continues to increase the number of beds for COVID-19 referral hospitals, from 310,700 hospital beds in 2019 to 430,000 in July 2021. All Indonesian hospitals follow the National Guideline (for diagnosis and therapy) protocols published at the relevant time alongside the period of patient admission. COVID confirmed patients were provided healthcare in the COVID referral hospitals with the government bearing all costs of COVID-19 treatments. It is also important to note that all hospitals in Indonesia may not have access to the conventional recommended contemporary drugs, and therefore treatment of patients may vary.

To date, there are limited evidence-based drugs that are effective against COVID-19 infection [4]. In October 2020, the FDA-approved remdesivir (100 mg injection) for treatment of COVID-19 due to its shorter time to recovery compared to placebo group [5,6]. The Adaptive COVID-19 Treatment Trial (ACTT-1) is a randomized, double-blind, placebo-controlled trial, with 1062 patients (541 assigned to remdesivir and 521 to placebo) [6]. Many drugs that were utilized to treat this disease were not antivirus drugs. Antibacterials and anti-inflammatory drugs were used due to their mechanism of action. Chloroquine, for example, interferes with lysosomal activity and autophagy, which results in inhibition of cytokine production and modulation of certain co-stimulatory molecules. It is widely used for the treatment of certain types of malaria and extraintestinal amebiasis. Azithromycin is a common Food and Drug Administration (FDA)-approved antibacterial used to treat many types of infections. It binds to the 23S rRNA of the bacterial 50S ribosomal subunit, which results in inhibition of bacterial protein synthesis. Lopinavir, a Human Immunodeficiency Virus (HIV) protease inhibitor, is also commonly used against COVID-19, and ritonavir is a potent inhibitor of the enzymes responsible for lopinavir metabolism; the combination of these two is a FDA-approved antiretroviral combination used to manage HIV positive patients. Dexamethasone is a well-known and potent glucocorticoid (a synthetic adrenocortical steroid), which inhibits neutrophil apoptosis and demargination, inhibits phospholipase A2, decreases the formation of arachidonic acid derivatives, inhibits NF-Kappa B, and promotes interleukin-10. It is mainly used for endocrine disorders, allergic states, and local treatment. On one hand, hydroxychloroquine, chloroquine, and azithromycin had been reported to have a small benefit on COVID-19 patients’ mortality [4]. On the other hand, a retrospective observational study reported that remdesivir and lopinavir/ritonavir usage were effective in reducing COVID-19′s viral load in patients [7,8]. A recent meta-analysis had also reported that corticosteroids reduce COVID-19′s progression and improve mortality rate for patients in both Intensive Care Units (ICU) and non-ICU compared to standard care [9].

However, there has been some reported adverse drug events related to medication use in COVID-19 patients. The risk of cardiac arrest, for example, was significantly higher in patients receiving both hydroxychloroquine and azithromycin (OR = 2.13) [10]. Another common adverse effect for lopinavir-ritonavir includes gastrointestinal disturbance (up to 28% patients), most notably diarrhea and nausea, in COVID-19 patients. Hepatotoxicity (2–10% patients) was also reported [11,12]. Early administration of a high dose of corticosteroids is associated with delayed viral clearance with increased incidence of severe bacterial infection and hypokalemia [13]. However, a prospective meta-analysis showed that the administration of systemic corticosteroids in critically ill patients was associated with a lower 28-day all-cause mortality rate than usual care or placebo [14].

Injudicious and excess use of antibacterials will lead to drug resistance problems in the future [15]. A review of studies published on hospitalized COVID-19 patients identified that while 72% (1450 out of 2010 individuals) of patients received antibacterials, only 8% (62 out of 806 individuals) demonstrated superimposed bacterial or fungal co-infections [15,16]. Azithromycin, a broad-spectrum macrolide antibacterial has become a common treatment for COVID-19 patients in several parts of the world although it is not yet recommended outside of COVID-19 clinical trials [17,18,19,20]. The World Health Organisation’s (WHO) interim guidance on the clinical management of COVID-19 does not recommend antibacterial therapy or prophylaxis for patients with mild or moderate COVID-19 unless signs and symptoms of a bacterial infection are present. It is recommended that daily assessment is performed on patients for the use of antibacterial treatment with bacterial coinfection [18]. The guidance further states that empiric antibacterial pneumonia treatment should be considered for older people residing in long-term care facilities and children younger than five years with moderate COVID-19 illness.

This paper seeks to contribute to the current growing body of knowledge of studies for COVID-19 treatments. We hypothesise that the use of antibacterials may not be as effective compared to antivirals in improving COVID-19 patients’ outcome and in shortening their length of stay. This study aims to explore the effectiveness of various antibacterial and antiviral medications administered in the management of patients with COVID-19.

## 2. Materials and Methods

### 2.1. Study Design

The minimum sample required in this study was calculated using the Lemeshow equation as the prevalence in the population is unknown [21,22], where a total of approximately 96 to 100 observations is required for each hospital sample (Equation (1)). The inclusion criteria for patients in this study was a confirmed positive PCR test from mBioCoV-19 RT-PCR Kit (PT Bio Farma, Bandung, Indonesia) alongside a positive diagnosis from a doctor. Patient outcome and length of stay were selected as the response variable as a positive outcome combined with the shortest length of stay in the hospital was desired to decrease the burden of the healthcare system.

(1) $$n=\frac{Z^{2}\times P\left(1-P\right)}{d^{2}}$$

*n* = number of required samples
*Z* = *Z* score (at 95% CI = 1.96)
*P* = population proportion (unknown, therefore set at 0.5)
*d* = alpha (0.10) (sampling error of 10%)

$$n=\frac{{\left(1.96\right)}^{2}\times 0.5\left(0.5-1\right)\;}{{\left(0.10\right)}^{2}}=96.04\;\sim \;100$$

### 2.2. Data Collection

The material for this research was medical records of hospitalized COVID-19 patients’ Rumah Sakit Bhayangkara (Surabaya), Rumah Sakit Pusat TNI Angkatan Laut (RSPAL) Dr. Ramelan (Surabaya), Rumah Sakit Umum (RSU) Haji (Surabaya), Rumah Sakit Umum Daerah (RSUD) Bangil (Pasuruan) for the period of July–December 2020. This dataset includes patients’ demographic data (e.g., gender and age), COVID-19 clinical spectrum based on National Institute of Health’s (NIH) COVID-19 Treatment Guideline [23]. The spectrum of illness can range from asymptomatic infection to severe pneumonia with acute respiratory distress syndrome and death. Mild defined as no pneumonia or mild pneumonia; severe defined as dyspnea, respiratory frequency ≥30 breaths/min, oxygen saturation [SpO2] ≤93%, a ratio of arterial partial pressure of oxygen to fraction of inspired oxygen [PaO2/FiO2] <300 mm Hg, and/or lung infiltrates >50% within 24 to 48 h); and critical defined as respiratory failure, septic shock, and/or multiorgan dysfunction or failure) (e.g., asymptomatic, mild, and moderate), comorbidities (e.g., diabetes, kidney disease), administered number of antibacterial and antiviral agents, type of antibacterial and antiviral administered, length of stay (in days), and COVID-19 outcome (as positive/negative outcome).

A categorization was made for comorbidity data, for example, indigestion and dyspepsia were categorized into digestive system comorbidity while emphysema and asthma were categorized into respiratory system comorbidity. Additional categorization was also carried out, especially for pneumonia induced by COVID-19 and the presence of diabetes in patients. Which antibacterials were used was also classified under WHO’s Anatomical Therapeutic Chemical (ATC) Classification System, which utilizes a classification system based on the active ingredients in accordance with the organ system or its therapeutic, pharmacological, and chemical properties. Antibacterials and antivirals in this study were classified up to the fourth (e.g., J01E) and fifth level (e.g., J05AH) of ATC respectively.

### 2.3. Drug Profile

In this study, defined daily dose of antibacterial showed the number of unnecessary antibacterials used for treatment of COVID-19 [24]. The number of drugs used was calculated as percentage and DDD (defined daily dose)/100 bed-days [25,26].

(2) $$\mathrm{DDD}/100\;\mathrm{bed}-\mathrm{days}=\frac{\mathrm{number}\;\mathrm{of}\;\mathrm{antibacterial}\;\left(\mathrm{gram}\right)}{{\mathrm{WHO}}^{\prime }\mathrm{DDD}\;\mathrm{antibacterial}\left(\mathrm{gram}\right)}\;\times \;\frac{100}{\mathrm{Length}\;\mathrm{of}\;\mathrm{Stay}\left(\mathrm{days}\right)}$$

### 2.4. Statistical Analysis

The multiple mixed-logit regression model was utilized to explore the effectiveness of various antibacterial and antiviral drugs administered in this study based on patients’ COVID-19 outcome. Additionally, patients’ age, gender, COVID-19 severity, and comorbidity were included as confounding factors. Automatic weigh correction was applied to the model as the frequencies between the two outcomes was unbalanced. Odds ratio (hereafter called ratio of cure) was also calculated for factors that reached significance in the model. In addition, the Analysis of Covariate (ANCOVA) model was employed to investigate which antibacterials and antivirals were significant in reducing the length of stay for COVID-19 patients with a positive outcome. Confounding factors (e.g., age, comorbidity) that were used in multiple mixed-logit regression were also employed in this model. Level of significance for all tests was set at 5% level. All statistical analyses in this study were carried out using XLSTAT 2021.4.1.1199 (Addinsoft, Paris, France).

## 3. Results

### 3.1. Demographics

This study is representative of several tertiary health facilities government ownership hospitals in Indonesia. Eighty percent of COVID-19 patients admitted to hospitals were aged 25–65 years, the number of mild-moderate patients was more than the number of severe patients, and the average length of stay was 8–13 days. Heart disease and diabetes were the most common comorbidities among COVID-19 patients’ (Table 1).

### 3.2. Drug Use Profile

The use of antibacterials in hospital D (201 DDD per 100 bed-days) was twice that of hospital A (87 DDD per 100 bed-days), hospital B (92 DDD per 100 bed-days), and hospital C (128 DDD per 100 bed-days). The most common antibacterial used was azithromycin (33–65 DDD per 100 bed-days) (Table 2). Oseltamivir was the most prescribed antiviral because it was recommended by the Indonesian guideline version 2 [27] when this study was conducted (Table 3).

### 3.3. Drug Effectiveness

The mixed-logit regression model revealed that the administration of various antibacterials and antivirals showed significant differences in regards to patients’ COVID-19 outcome (*Wald X*^2^_(20)_ = 101.72; *p <* 0.001) and that variables in the model brought a significant amount of information (*Log Ratio X*^2^_(20)_ = 185.12; *p* < 0.001).

The model revealed that administration of beta-lactam antibacterials (J01D) (OR: 3.10 (0.961–9.39 at 95% CI)) and favipiravir (J05AX) (OR: 6.82 (0.98–47.32 at 95% CI)) was a positive significant factor in improving patients’ COVID-19 outcome (Table 4).

Age (*β* = 0.315; *p <* 0.001), COVID-19 induced pneumonia (*β* = 0.412; *p <* 0.001), heart (*β* = 0.215; *p <* 0.01), respiration (*β* = 0.280; *p <* 0.01), liver (*β* = 0.224; *p <* 0.05), and other (i.e., obesity and cancer) (*β* = 0.157; *p <* 0.05) comorbidities were shown to be significant confounding factors in the model (Table 5). It is very important to note that only three patients had the classification of ‘liver’ and only two had the classification ‘others’, only two patients were reported to have obesity, and only one patient was reported to have cervical and lung cancer in this study.

### 3.4. Length of Stay

A significant difference was observed using the ANCOVA model (Table 6) for the length of stay for successful treatment of COVID-19 patients who varied in their antibacterial and antiviral treatment (*F*_(18,375)_ = 2.71; *p <* 0.001). More specifically, tenofovir (J05AF) (*F* = 4.71; *p <* 0.05; Δ*μ* = 2.38), and favipiravir (J05AX) (*F* = 7.21; *p <* 0.001; Δ*μ* = 1.79) showed a significant and meaningful decrease in the length of stay of patients with successful treatment outcome by more than 1 day. It is however important to note that the use of tenofovir was only reported for one patient.

To no surprise, significant confounding factors that influenced length of stay were COVID-19 clinical spectrum (*F* = 2.18; *p <* 0.05; Δ*μ*_(severe–asymptomatic)_ = 1.62) and respiration comorbidity *(F* = 6.34; *p <* 0.01; Δ*μ* = 1.81) (Table 7).

## 4. Discussion

### 4.1. Increased Use of Antibacterials and Antivirals during COVID-19 Pandemic

The antibacterials for COVID-19 patients in this study were generally overused. In this study, antibacterials were prescribed for almost all patients with COVID-19 with 17 types of antibacterials used by more than 90% of patients, resulting in 87 to 201 DDD per 100 bed-days. A recent review reported a rate of antibiotic use of 54% to 68% for non-severe patients and 80% to 100% for severe patients with COVID-19, with only 18% of patients who received antibiotics having secondary (14%) or co-infections (4%) [27]. Fluoroquinolone, azithromycin [30,31], and ceftriaxone [24,30,32] were the most commonly prescribed antibacterials [33]. In this study, azithromycin (macrolide antibacterial) was the most prescribed antibacterial, following COVID-19 management published by the Indonesian Ministry of Health [27]. In addition, thirteen out of seventeen antibacterials used were antibacterials in Watch categories [28,29]. Importantly, this may lead to higher resistance potential. Antibiotic selection pressure [34,35] would increase the incidence of multidrug-resistant organisms. Thirteen bacteria have been identified as causing a secondary infection to SARS-CoV-2, where it may lead to increased mortality and morbidity for COVID-19 patients [36].

SARS-CoV-2 is an enveloped positive-strand RNA virus [37]. Any antiviral drug with a mechanism of action targeting the RNA genome would potentially eliminate viruses from the host. Remdesivir, the first nucleoside analog approved by the FDA for hospitalized COVID-19 patients’ [38], inhibits the RNA-dependent RNA polymerase (RdRp) of coronaviruses. Aside from developing a new drug, researchers have repurposed some nucleoside analogue reverse transcriptase inhibitors (NRTIs) for SARS-CoV-2 infection [39]. These NRTIs include zidovudine (AZT), tenofovir disoproxil fumarate (TDF), tenofovir alafenamide (TAF), abacavir (ABC), and stavudine (d4T), which can potentially be useful against COVID-19 infection [40].

### 4.2. Antiviral Treatment Showed a Positive COVID-19 Outcome and Decreased Length of Stay

This study showed a benefit of both antibacterials and antivirals in improving patients’ COVID-19 outcome and shortening their length of stay. Interestingly, the clinical benefit of antiviral treatment was reported to be inconclusive. Some trials showed a better therapeutic response [41], but others showed no clinical benefit [42,43,44]. Reported adverse effects include diarrhea [45], hyperuricemia [42], and elevated transaminases (up to more than 5 times the upper limit of normal) [46].

Favipiravir both significantly improved patients’ COVID-19 outcome and decreased their length of stay in our study. As mentioned above, this is because favipiravir’s mechanism of action inhibits the RNA-dependent RNA polymerase (RdRp) of RNA viruses [47]. Other studies showed similar results, where COVID-19 patients’ viral clearance rate of favipiravir was reported at 4 days [48] with its clinical recovery rate at day 14 more than 80% [49,50].

Although tenofovir in this study reached significance, this was only reported for one patient. A previous review has discussed repurposing nucleotide analogue reverse-transcriptase inhibitors for SARS-CoV-2 infection (i.e., tenofovir, abacavir, emtricitabine, zidovudine, particularly tenofovir). Tenofovir was the first nucleotide analogue reverse-transcriptase inhibitor approved by the Food and Drug Administration (FDA) in October 2001. It has been marketed in the United States for the management of HIV infection. The cost of tenofovir per day is only 0.2% the price of remdesivir. There are currently eight ongoing clinical trials investigating the efficacy of tenofovir for COVID-19 treatment (6 trials) and prevention (2 trials) [39].

It is generally understood that antibacterials are not effective against infection caused by viruses. Interestingly, our results showed that beta-lactam antibacterials improved patients’ COVID-19 outcome but not the length of stay. This was perhaps due to β-lactams tolerability and efficacy against many Gram-positive and Gram-negative bacteria that cause secondary infection [36] in COVID-19 patients. To date, there are no studies that report on its effectiveness in COVID-19 patients [51]; previous studies have only reported its prescription rate, which ranges from 9 to 40% [30,52] and its duration of treatment (e.g., 1 g twice daily for 7 days) [30]. Resonating with WHO’s recommendation, it is recommended that health practitioners perform an assessment for co-infections. We speculate that patients in our study may have had secondary bacterial co-infection and benefited from the administration of antibacterials.

### 4.3. Comorbities Increase the Mortality Rate and Length of Stay of Patients with COVID-19

Age was a significant demographic effect that influenced COVID-19 outcome. It was reported that the older population in this study showed a higher risk of a negative COVID-19 outcome compared to the younger population. Our results resonate with a cohort retrospective study in 55 hospitals in Jakarta, where 70% of deceased COVID-19 patients were older than 50 years old [3].

In general, the mortality risk of COVID-19 patients with comorbidities had been reported to be higher than patients without comorbidities [3,53,54,55]. A study of severe COVID-19 pneumonia showed that of 55% deceased patients, 70% of them had reported having comorbidities (e.g., hypertension, diabetes, ischemic heart disease) [56]. Another cohort retrospective study of hospitalized COVID-19 patients had reported that 32% of deceased patients had pneumonia [57]. Although relatively high, this number was lower than patients with severe COVID-19 pneumonia [56]. Their results were similar to our study where COVID-19 pneumonia was a stronger predictor of COVID-19 recovery compared to existing respiratory comorbidity.

Hypertension and diabetes were the most commonly reported comorbidities among inpatients with COVID-19 [3,53,58]. In this study, 21% of patients with COVID-19 were reported to have heart problems, which was also found to be a significant predictor for COVID-19 recovery. Interestingly, 18% patients with diabetes in this study did not reach statistical significance as a predictor of COVID-19 recovery. A meta-analysis reported that there is a three times higher mortality risk in patients with cardiovascular disease; and two times higher mortality risk in patients with diabetes [53]. Cardiovascular disease and diabetes were consistently reported as a significant predictor for mortality [59]. Although in rather small numbers, other comorbidities have been reported, such as liver damage, obesity and cancer, and they were found to be a significant predictor of COVID-19 recovery. Several studies have reported that obesity was associated with an increased risk of death from COVID-19 [60,61,62,63], particularly in those aged more than 65 years [61].

The length of stay of COVID-19 patients with severe illness is significantly higher than moderate illness. The median duration of hospitalization ranged from 5 to 29 days [64]. Echoing other studies, our study showed an average length of stay of 13 days, and that mortality of patients with severe disease was higher than those with non-severe disease. Another study reported that the 28-day mortality of the severe group (SpO2 < 90%) was six times higher than the moderate group (SpO2 > 90%) [65], and higher Sequential Organ Failure Assessment (SOFA) scores were also correlated with higher mortality rate [66].

In our study, six percent of patients had respiration impairment, and respiration was the only comorbidity that influenced length of stay, with a mean difference of 1.8 days. During the COVID-19 pandemic, the prevalence of the acute respiratory diseases asthma and chronic obstructive pulmonary disease (COPD) were low [67,68,69]. This could be related to a wide variety of factors, including effective lockdowns, better quality of air, and regular use of masks, which were probably responsible for the reduced incidence of exacerbations of these chronic respiratory diseases [70]. Moreover, systematic reviews have shown an unclear relationship between respiratory impairment, risk of infection, and severe outcome from COVID-19 infection [68]. Further study to explain this result is required.

## 5. Conclusions

This study demonstrates that various drugs have been used for the treatment of COVID-19. The most prescribed antibacterial and antiviral drugs in this study were in adherence to the local Indonesian National Guideline where the study was conducted. This study showed that favipiravir improved patients’ COVID-19 outcomes, but not azithromycin and oseltamivir. Based on this evidence and echoing the recommended guidelines, we recommend administration of favipiravir to improve patients’ COVID-19 outcome and decrease their length of stay in accordance with Indonesia’s new treatment guideline in January 2022. It is however important to note that this study is an observational study and that the drugs used are yet to be proven effective for COVID-19 in a randomized clinical trial manner. Additionally, this study utilized historical medical data provided by the hospital, which means that the analysis of subgroups may be too small to reach reliable conclusions. This study also warrants future research to develop potentially new drugs or repurpose old drugs to combat COVID-19.

## Figures and Tables

**Table 1:** Baseline demographic inpatient COVID-19.

Variable	RS A (N = 94)	RS B (N = 92)	RS C (N = 100)	RS D (N = 146)
Hospital Ownership	Government (Police)	Government (Navy)	Government	Government
Hospital type †	B	B	B	B
Number of beds	234	692	225	366
Gender				
Male	58 (62%)	58 (63%)	61 (61%)	72 (49%)
Female	36 (38%)	34 (37%)	39 (39%)	74 (51%)
Age (years)				
0–5	1 (1%)	0	0	0
5–11	1 (1%)	0	0	0
12–16	3 (3%)	0	0	0
17–25	32 (34%)	3 (3%)	0	13 (9%)
25–45	36 (38%)	21 (23%)	27 (27%)	59 (40%)
45–65	21 (23%)	61 (66%)	57 (57%)	63 (43%)
>65	0	7 (8%)	16 (16%)	11 (8%)
Clinical spectrum				
Asymptomatic	5 (5%)	0	0	0
Mild	46 (49%)	0	0	0
Moderate	43 (46%)	58 (63%)	81 (81%)	59 (60%)
Severe	0	34 (37%)	19 (19%)	87 (40%)
Length of Stay (mean, SD)				
Asymptomatic	9.6 (3.1)	0	0	0
Mild	11 (4.8)	0	0	0
Moderate	12.7 (4.4)	12.6 (5.2)	11.1 (5.2)	8.9 (4.1)
Severe	0	8.9 (5.6)	8.7 (5.1)	9 (3.7)
Moderate and Severe	12.7 (4.4)	11.2 (5.6)	10.7 (5.2)	9 (3.8)
Comorbidity				
COVID induced Pneumonia	0	92 (100%)	100 (100%)	48 (32.9%)
Heart	9 (9.6%)	37 (40.2%)	28 (28%)	15 (10.3%)
Diabetes	2 (2.1%)	28 (30.4%)	24 (24%)	24 (16.4%)
Digestion	4 (4.3%)	4 (4.3%)	21 (21%)	8 (5.5%)
Respiration	1 (1.2%)	0	18 (18%)	9 (6.2%)
Blood	0	13 (14.1%)	25 (25%)	5 (3.4%)
Immune	0	0	1 (1%)	27 (18.5%)
Nerve	2 (2.1%)	3 (3.3%)	6 (6%)	2 (1.2%)
Kidney	0	0	2 (2%)	0
Liver	0	0	2 (2%)	1 (0.7%)
Obesity	0	3 (3.3%)	0	0
Cancer	0	3 (3.3%)	0	0
Skin	0	0	1 (1%)	0
Others	0	0	6 (6%)	1 (0.7%)

† In Indonesia, there are three classifications of health facilities (primary, secondary, tertiary), and four types of the hospital (A, B, C, D). Tertiary health facilities are referrals for secondary health facilities, and secondary health facilities are referrals for primary health facilities. Tertiary health facilities consist of hospital type A and B. Secondary health facilities consist of hospital type C and D. Primary health facilities are primary health care.

**Table 2:** DDD per 100 bed-days antibacterials.

Group	Name	ATC Code	RS A	RS B	RS C	RS D
Access *						
Penicillin beta-lactam (J01C)	ampicillin	J01CA01	0	0	0.2	0
	ampicillin and sulbactam	J01CR01	0	0.3	0.2	0
Aminoglycoside (J01G)	amikacin	J01GB06	0	2.1	0	0
Imidazole (J01XD)	metronidazole	J01XD01	0	0.6	0	0
Watch *						
Other beta-lactam (J01D)	cefuroxime	J01DC02	0	0	0	0.2
	cefditoren	J01DD16	0	0	0	0.2
	ceftazidime	J01DD02	0	1.9	0	1.8
	ceftriaxone	J01DD04	0	0.1	13.5	12.6
	cefixime	J01DD08	0	0	0.2	60.6
	cefoperazone	J01DD12	0	0.02	0	0
	cefoperazone and sulbactam	J01DD62	2.9	0.7	0	0
	cefepime	J01DE01	0	0.1	0	0
	meropenem	J01DH02	0.5	2.9	1.3	18.4
Macrolide (J01FA)	azithromycin	J01FA10	55	64.2	65.3	33
Quinolone (J01M)	ciprofloxacin	J01MA02	0	0.5	1.9	0
	levofloxacin	J01MA12	23.9	17.6	45	12.2
	moxifloxacin	J01MA14	4.3	0.7	0	61.7
Total			86.6	91.7	127.6	200.7

* The WHO’s AWaRe (access, watch, reserve) classification of antibiotics categorized antibiotics to the following: (1) Access group antibiotics that have activity against a wide range of commonly encountered susceptible pathogens, (2) Watch group antibiotics that have higher resistance potential, and (3) Reserve group antibiotics that were antibiotics of last resort when all alternatives have failed or are not suitable [28,29].

**Table 3:** Antivirals use during hospitalization.

Name	ATC Code	RS A (N = 94)	RS B (N = 92)	RS C (N = 100)	RS D (N = 146)
Remdesivir	J05AB16	6 (6.4%)	7 (7.6%)	0	49 (33.6%)
Tenofovir disoproxil	J05AF07	0	0	1 (1%)	0
Efavirenz	J05AG03	0	0	1 (1%)	0
Oseltamivir	J05AH02	4 (4.3%)	0	94 (94%)	107 (73.3)
Lamivudine, zidovudine	J05AR01	0	0	1 (1%)	0
Lopinavir, ritonavir	J05AR10	40 (42.6%)	86 (93.5)	3 (3%)	10 (6.8)
Favipiravir	J05AX27	43 (45.7%)	0	1 (1%)	31 (21.2)
Total		93 (99%)	93 (101.1%)	101 (101%)	197 (134.9%)

**Table 4:** Antibacterials and antivirals class and its effectiveness in improving patients’ outcome during hospitalization.

Antibacterials and Antivirals Class	Level of Significance	Odds Ratio (Lower-Upper Bound at 95%)
J01C	n.s.	-
J01D	<0.001	3.006 (0.962–9.397)
J01E	n.s.	-
J01F	n.s.	-
J01G	n.s.	-
J01M	n.s.	-
J01X	n.s.	-
J05AB	n.s.	-
J05AF	n.s.	-
J05AG	n.s.	-
J05AH	n.s.	-
J05AR	n.s.	-
J05AX	<0.001	6.820 (0.983–47.323)

**Table 5:** Patients’ confounding factors and their relation to hospitalization outcome.

Confounding and Comorbidity Factors	Standardised Coefficients	Level of Significance
Demographics		
Gender	-	n.s.
Age	0.315	<0.001
Comorbidities		
COVID induced Pneumonia	0.412	<0.001
Heart	0.215	<0.01
Diabetes	-	n.s.
Digestion	-	n.s.
Respiration	0.280	<0.01
Blood	-	n.s.
Liver	0.224	<0.05
Others	0.157	<0.05

**Table 6:** Antibacterials and antivirals class and its effectiveness in decreasing patients’ length of stay during hospitalization.

Antibacterials and Antivirals Class	Level of Significance	Difference of Length of Stay
J01C	n.s.	-
J01D	n.s.	-
J01E	n.s.	-
J01F	<0.05	0.44
J01G	n.s.	-
J01M	n.s.	-
J01X	n.s.	-
J05AB	n.s.	-
J05AF	<0.05	2.38
J05AG	n.s.	-
J05AH	<0.05	0.17
J05AR	n.s.	-
J05AX	<0.001	1.79

**Table 7:** Patients’ confounding factors and their relation to length of stay.

Confounding and Comorbidity Factors	Level of Significance	Difference in Length of Stay
Demographics		
Gender	n.s.	-
Age	n.s.	-
COVID-related factors		
COVID clinical spectrum	<0.05	1.62
COVID induced Pneumonia	n.s.	-
Comorbidities		
Heart	n.s.	-
Diabetes	n.s.	-
Digestion	n.s.	-
Respiration	<0.01	1.81
Blood	n.s.	-
Liver	n.s.	-
Others	n.s.	-

## Data Availability

The authors confirm that the data supporting the findings of this study are available on request.
